# Cholangioscopy-guided holmium laser ablation for stent-related mucosal hyperplasia causing recurrent cholangitis after endoscopic ultrasound-guided hepaticogastrostomy

**DOI:** 10.1055/a-2921-6146

**Published:** 2026-08-27

**Authors:** Tomoya Takahashi, Ko Tomishima, Yusuke Takasaki, Yoko Iwabe, Yusuke Yamaguchi, Daishi Kabemura, Hiroyuki Isayama

**Affiliations:** 1 Department of Gastroenterology 12847 Graduate School of Medicine, Juntendo UniversityTokyoTokyoJapan; 2 Division of GastroenterologyDepartment of Medicine 12737 Chiba UniversityChibaJapan; 3 Division of GastroenterologyDepartment of Medicine 26683 Chulalongkorn UniversityBangkokThailand

## Acronyms and abbreviations



**Video 1** A SpyGlass DS direct cholangioscopic view showing holmium laser ablation (0.9–1.0 J, 5 Hz) applied to hyperplastic tissue at the uncovered portion of the SpringStopper stent.

EUS-HGS: EUS-guided hepaticogastrostomy
PCSEMS: partially covered self-expandable metal stent
RBO: recurrent biliary obstruction
RFA: radiofrequency ablation

**Fig. 1:**
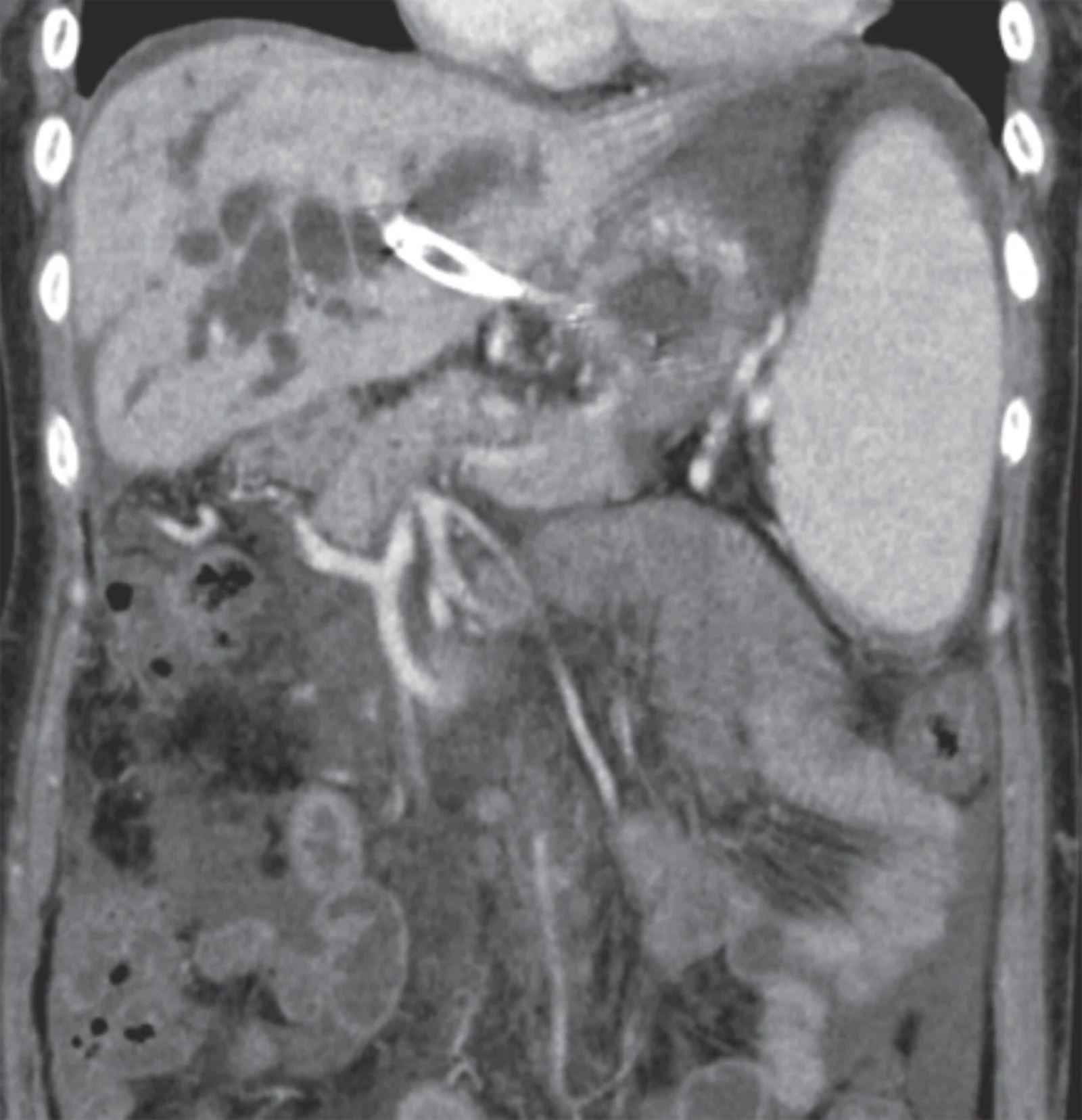
A coronal CT image showing intrahepatic bile duct dilat`ation proximal to the metal stent, suggesting obstruction due to mucosal hyperplasia.

**Fig. 2:**
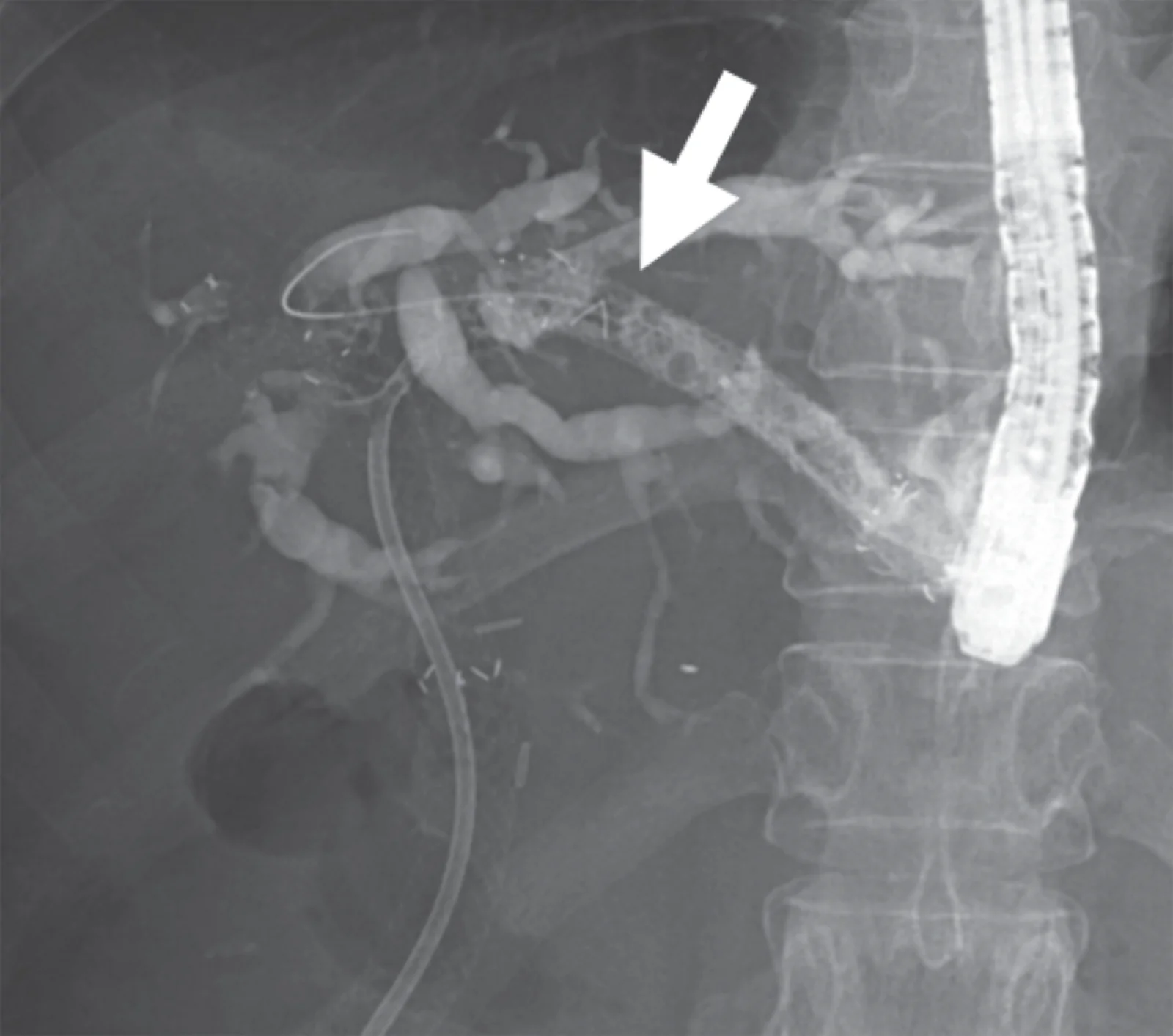
A fluoroscopic cholangiogram before laser ablation demonstrating a stricture at the hepatic side of the SpringStopper stent (arrow).

**Fig. 3:**
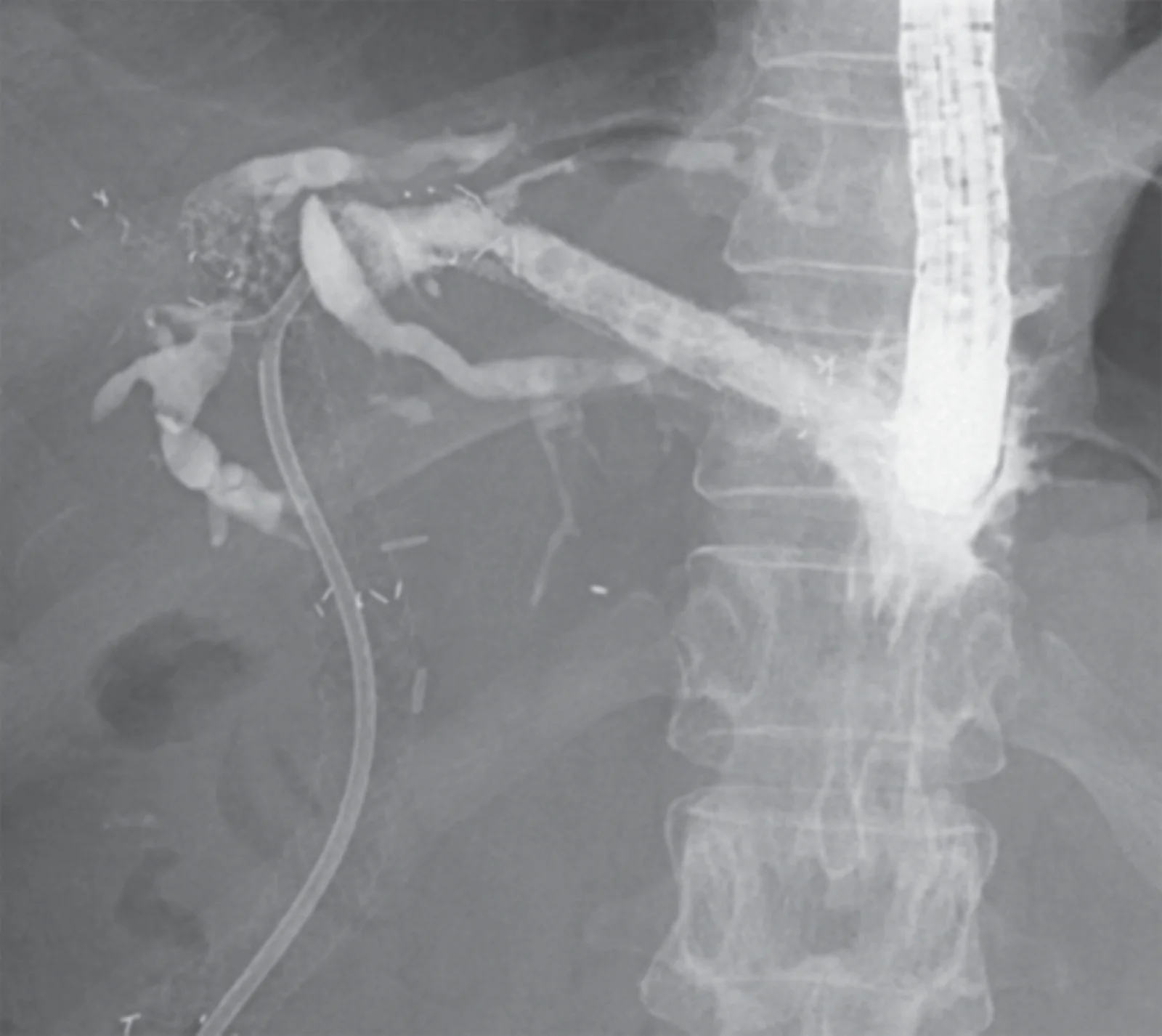
A fluoroscopic cholangiogram after laser ablation showing no residual filling defects and improved bile flow.

Endoscopic ultrasound-guided hepaticogastrostomy (EUS-HGS) is an established biliary drainage technique when transpapillary biliary access is insufficient. Recurrent biliary obstruction (RBO) after EUS-HGS is not uncommon; Nakai et al reported hyperplasia at the uncovered portion as the major cause after long partially covered metal stent placement. 1​ Reintervention strategies include radiofrequency ablation (RFA) 2​ , piercing techniques 3​ , and cholangioscopy-guided laser ablation. 4​ ​5​ However, laser ablation has not been applied to hyperplasia via the EUS-HGS route. A 53-year-old woman underwent EUS-HGS targeting B3 with simultaneous endoscopic retrograde cholangiopancreatography for the right hepatic duct. An 8-mm, 10-cm SpringStopper stent (Taewoong Medical, Gyeonggi-do, Korea) was placed. Three months later, hyperplasia-related cholangitis developed, and an 8-mm, 6-cm Zilver stent (Cook Medical, Bloomington, IN, USA) was added within the SpringStopper. Four months thereafter, cholangitis recurred. Coronal computed tomography showed intrahepatic bile duct dilatation proximal to the stent, suggesting obstruction ( Fig. 1 ). Cholangiography confirmed a stricture at the hepatic side ( Fig. 2 ). A guidewire was advanced through the hyperplastic tissue into the proximal bile duct. SpyGlass DS (Boston Scientific, Marlborough, MA, USA) was introduced, and holmium laser ablation was performed under direct cholangioscopic vision ( Video 1 ). Energy was initiated at 0.9 J/5 Hz and increased to 1.0 J until luminal breakthrough. Dilation was performed using an 8-mm REN balloon catheter (Kaneka, Osaka, Japan). Post-ablation cholangiography showed no residual filling defects ( Fig. 3 ). Cholangitis resolved without adverse events, with no RBO at 2 months. This case demonstrates holmium laser ablation for benign hyperplastic RBO after EUS-HGS. Because RFA provides circumferential thermal ablation and may be less suitable for selectively fenestrating a membranous hyperplastic stricture, cholangioscopy-guided laser ablation may offer a more controlled approach for focal recanalization. However, the procedure was time consuming (100 min), and careful patient selection is warranted.

Endoscopy_UCTN_Code_TTT_1AR_2AL

## References

[1] Nakai Y Sato T Hakuta R Long-term outcomes of a long, partially covered metal stent for EUS-guided hepaticogastrostomy in patients with malignant biliary obstruction (with video) Gastrointest Endosc 2020 92 623 631 32278705 10.1016/j.gie.2020.03.3856

[2] Matsubara S Nakagawa K Suda K Radiofrequency ablation of hyperplasia at an uncovered portion of a partially covered metal stent in endoscopic ultrasound-guided hepaticogastrostomy (with video) J Hepatobiliary Pancreatic Sci. 2021 28 e32 e33 10.1002/jhbp.1004 34057821

[3] Otsuka Y Minaga K Hara A Piercing technique for mucosal hyperplasia at an uncovered part of a partially covered stent after endoscopic ultrasound-guided hepaticogastrostomy Endosc Int Open 2023 11 E811 E813 10.1055/a-2132-5116 37664787 PMC10473886

[4] Takasaki Y Isayama H Yamaguchi Y Peroral chola ngioscopy-guided laser-stricturoplasty for intrahepatic benign biliary strictures (with video) Gastrointest Endosc 2026 103 799 803 10.1016/j.gie.2025.10.024PMID:41115476 41115476

[5] Enke T Rueb N Pessorrusso F Han S Shah R J Long-term follow-up of patients undergoing cholangiopancreatoscopy-guided laser dissection and ablation for refractory pancreatic and biliary strictures (with video) Gastrointest Endosc 2026 103 02 347 354 10.1016/j.gie.2025.08.059PMID:40934982 40934982

